# Vehicle Localization Kalman Filtering for Traffic Light Advisor Application in Urban Scenarios

**DOI:** 10.3390/s23156888

**Published:** 2023-08-03

**Authors:** Daniele Vignarca, Stefano Arrigoni, Edoardo Sabbioni

**Affiliations:** Department of Mechanical Engineering, Politecnico di Milano, 20156 Milan, Italy; daniele.vignarca@polimi.it (D.V.); stefano.arrigoni@polimi.it (S.A.)

**Keywords:** vehicle localization, Kalman filter, ADAS, kinematic model, GPS, TLA, ITS

## Abstract

The recent advancements in Intelligent Transportation Systems (ITS) have revealed significant potential for enhancing traffic management through Advanced Driver Assist Systems (ADASs), with benefits for both safety and environment. This research paper proposes a vehicle localization technique based on Kalman filtering, as accurate positioning of the ego-vehicle is essential for the proper functioning of the Traffic Light Advisor (TLA) system. The aim of the TLA is to calculate the most suitable speed to safely reach and pass the first traffic light in front of the vehicle and subsequently keep that velocity constant to overcome the following traffic light, thus allowing safer and more efficient driving practices, thereby reducing safety risks, and minimizing energy consumption. To overcome Global Positioning Systems (GPS) limitations encountered in urban scenarios, a multi-rate sensor fusion approach based on the Kalman filter with map matching and a simple kinematic one-dimensional model is proposed. The experimental results demonstrate an estimation error below 0.5 m on urban roads with GPS signal loss areas, making it suitable for TLA application. The experimental validation of the Traffic Light Advisor system confirmed the expected benefits with a 40% decrease in energy consumption compared to unassisted driving.

## 1. Introduction

Vehicle localization represents a fundamental task in many fields, ranging from Autonomous Vehicles (AV) to Advanced Driving Assistance Systems (ADASs) as well as traffic management [1]. Indeed, the starting point for most of the control logic, on both the vehicle and infrastructure sides, is the knowledge of the vehicle position. This is typically fed to algorithms intended to compute either vehicle optimal trajectory and speed or safety risk indexes to safely overcome dangerous situations, especially in urban scenarios.

Vehicle localization techniques can be distinguished between the onboard sensor-based systems and those relying upon Vehicle-to-Vehicle (V2V) and Vehicle-to-Infrastructure (V2I) communication. The former category can be further split into active sensor based (e.g., LiDAR and RADAR) and passive sensor based (e.g., GPS and IMU). On the one hand, active sensors are generally more costly and computationally expensive than passive ones. On the other hand, Inertial Measurement Units (IMUs) suffer from noisy signals which may lead to integration divergence, while when dealing with Global Positioning Systems (GPS) a typical issue is the signal loss [2]. In vehicle localization, GPS outage is thus a relevant phenomenon to cope with, especially in urban scenarios, where the presence of trees and high buildings limit the sensor capabilities.

The Green Light Optimal Speed Advisory (GLOSA) system is an application that conveys speed references to the driver to achieve lower travel times, fuel/energy consumption, and safer travel conditions [3]. This can be achieved thanks to the knowledge of road data, the vehicle state in terms of position and speed, and traffic light schedules. The speed profile calculation is typically addressed by taking into account different criteria. One typical approach is to minimize the engine power demand and idling time [4]. Another approach prioritizes driver annoyance reduction by minimizing the difference between the suggested and actual speeds, or by aiming to pass the traffic light in the smallest amount of time [5].

In this framework, the present study proposes a multi-sensor multi-rate vehicle localization technique based on Kalman filtering as the ego-vehicle position is a prerequisite for making ADASs work. The aim is to have an accurate state estimation that provides the vehicle position to the Traffic Light Advisor (TLA) system thought to be used in challenging scenarios such as urban roads, where pure GPS information may be neither present nor reliable. The field tests for the localization algorithm demonstrated good accuracy results in different conditions and GPS signal availability, proving to be consistent for running ADASs, such as the TLA. Furthermore, having implemented the localization algorithm and knowing the working plans of a set of traffic lights on a predefined path of the city of Milan, the paper reports full-scale testing on a trolley-bus in the urban scenario of the TLA developed by [6] at Politecnico di Milano, confirming the previously obtained simulation results.

The remainder of this paper is structured as follows: After the literature review in Section 2, the experimental setup used for the experimental campaigns is presented in Section 3. Section 4 details the proposed localization algorithm, and Section 5 summarizes the main feature of the TLA and presents the scenarios and the metrics adopted for the ADAS validation. The results of both the localization algorithm and the TLA system are reported in Section 6 while Section 7 draws the conclusions of this work, proposing some future development for the implemented systems.

## 2. State of the Art

In the literature, the criteria employed for the determination of the speed profile in GLOSA applications typically involve the minimization of the total energy consumption and travel time [7,8,9,10]. This kind of ADAS can be further distinguished into two categories based on the number of traffic lights they consider in real time to determine the recommended speeds: single-segment GLOSA (S-GLOSA) and multiple-segment GLOSA (M-GLOSA). S-GLOSA systems focus on analyzing the first traffic light encountered by the vehicle, while M-GLOSA systems consider multiple traffic lights along the vehicle’s route. In the case of S-GLOSA algorithms, they typically employ modeling approaches incorporating velocity profiles both upstream and downstream of the intersection, as demonstrated in [11]. However, in recent years, data-driven approaches have emerged as a promising alternative, as highlighted in [12]. In that research, a conventional S-GLOSA system is contrasted with reinforcement learning (RL) implementation, which incorporates data from a single traffic light and limited information from the preceding three vehicles. The RL-based approach resulted in an 11% increase in energy savings compared to the standard S-GLOSA system. These developments in data-driven methodologies highlight the potential to enhance the performance and energy efficiency of GLOSA systems.

In the future, Vehicle-to-Infrastructure (V2I) communication will be one of the main drivers making this kind of Advanced Driving Assistance System possible by providing a great amount of data about adjacent vehicles and traffic [13]. In fact, the work in [14] analyzes the effect of the GLOSA system running different simulations varying both infrastructure variables (e.g., cycle times and communication range) and external variables, such as traffic conditions. As far as traffic is concerned, the research conducted in [15] demonstrates the effectiveness of M-GLOSA systems compared to single-segment approaches, especially in free-flow traffic conditions. However, optimizing M-GLOSA systems while considering traffic light phase changes presents challenges, leading to non-convex feasible solution domains. To tackle this issue, the literature proposes various approaches. Studies like [8,15] have implemented Genetic Algorithms (GAs) to address the optimization problem. Additionally, search-based algorithms, employing semi-heuristic or brute-force methods, have also been explored in [11]. A widely adopted alternative is Model Predictive Control (MPC), and the authors in [16] investigated the MPC application for GLOSA implementation in road segments containing multiple traffic lights.

Self-localization has been extensively studied in the literature for a long time, as it serves as a crucial component in the development of Cooperative Active Safety Systems and ADASs in general. In a comprehensive review conducted in [17], various sensor-based and communication-based approaches for localization are thoroughly examined, with a specific focus on accuracy and real-time performance. The findings of the survey show that data fusion techniques, such as the integration of onboard passive sensors and Vehicle-to-Everything (V2X) communication, offer a promising solution due to their robustness, accuracy, and ability to operate in real time.

GPS outages represent a huge limiting point in vehicle localization in urban canyoning; thus, the most adopted strategy to cope with this issue is to use the Kalman filter and its variants to estimate the vehicle state also when GPS is not available, fusing different sensors (e.g., Inertial Measurement Unit and Wheel Speed Sensor) and a dynamic vehicle model [18,19]. In [2], an extended Kalman filter (EKF) fusing a digital map, IMUs, GPS data, and cellular Base Transceiver Stations (BTS) signals is presented. In their work, the authors proposed the use of cellular BTS not only to overcome GPS outages but also to improve localization accuracy.

Recently, LiDAR and vision sensors have been adopted to overcome the challenges of localization in urban scenarios, fusing these types of sensors to cope with the limitation of every single device [20]. In [21], a cascading Kalman filter and dynamic object removal model using multi-GNSS, INS, Precise Point Positioning (PPP), and vision to improve vehicle navigation performances in urban scenarios is presented. In this framework, a novel application in state estimation is Simultaneous Localization and Mapping (SLAM) which can be involved either in AV applications or in Cooperative ITS. On the one hand, Bersani et al. [22] presents an integrated system for vehicle state estimation using unscented Kalman filter fusing data from different passive sensors, such as GPS and IMUs, and from active sensors, like RADAR and LiDAR, which are used to detect and track obstacles as well as improve the localization algorithm. On the other hand, Wang et al. [23] compares different localization systems based on both GNSS and V2X communication for inter-vehicle distance calculation, which is needed for safety ADAS applications.

An interesting challenge in fusing different sensors is dealing with their different sampling frequency. In fact, in asynchronous multi-sensor systems, there is the possibility to miss some data when performing state estimation [24]. The authors in [25] presented different multi-rate multi-sensor models for Kalman filtering with missing measurements. The idea is to run the state estimation algorithm at the fastest sensor frequency and just predict the state vector whenever a sensor is missing or it is considered not reliable.

In addition to the widely studied Kalman filtering techniques, which are extensively covered in the vehicle localization literature, alternative strategies like graph optimization have also been explored. The authors in [26] introduced a novel approach in their work, presenting a multi-sensor fusion method formulated as a graphical model. This model optimizes the integration of onboard sensors to enhance positioning performance, utilizing a kinematic vehicle model as the underlying basis.

Recently, the advent of the 5G network cooperative oriented the research toward new horizons, such as cooperative localization and the Internet of Vehicles (IoV). In fact, thanks to the perception algorithm of the surrounding vehicles, vehicle communication allows gathering localization information that can be used to either improve the ego-vehicle state estimation when GPS is not accurate [27] or to fill the gap in case the GPS signal is missing for long periods [28]. Another approach investigated in the literature in the last decades is the use of Assisted-GPS (A-GPS) systems which exploit the terrestrial communication link to determine the current location [29]. This type of localization is typically employed on cell phones to avoid decoding the GPS messages for each satellite observed, thus using a remote server [30]. More recently, an alternative A-GPS system combining a barometer and accelerometer is proposed in [31] to improve the localization on smartphones.

This research paper introduces a localization technique that is both simple and reliable, offering robustness and accuracy for a TLA application. The approach relies on the use of a Kalman filter and map matching. The filter incorporates a one-dimensional uniformly accelerated kinematic motion model and integrates data from various onboard sensors, including the IMU, GPS, and Electronic Control Unit (ECU), each operating at different frequencies. Moreover, the localization algorithm is integrated into the Traffic Light Advisor (TLA) system, as developed in [6]. This integration optimizes the vehicle’s speed, minimizing unnecessary stops and ensuring a smoother driving experience, ultimately reducing energy consumption.

The primary contribution of this study is the development and full-scale experimental testing of a localization algorithm in urban scenarios facing GPS signal loss conditions. In particular, the algorithm utilizes multiple sensors and different sampling rates, making it suitable for implementing Advanced Driver Assistance Systems (ADAS), including Traffic Light Advisor (TLA) systems. The research mainly focuses on the urban road environment, where traditional GPS systems exhibit poor performance due to the challenges posed by urban canyoning. Instead of relying solely on velocity integration, the algorithm leverages the combined information from the Electronic Control Unit (ECU) for speed, Inertial Measurement Unit (IMU) for longitudinal acceleration, and GPS measurements to achieve a smoother output and accurate vehicle localization even in the absence of reliable GPS data. Alongside the presentation of the localization algorithm, the study also showcases the practical application of the proposed method. Specifically, experimental results for the Traffic Light Advisor (TLA) system are presented to further validate the approach introduced in [6] through real-world road tests.

## 3. Experimental Setup

**Figure 1:**
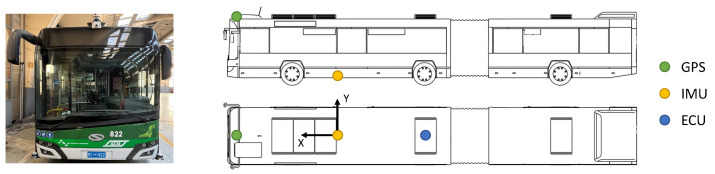
View and schematic representation of the instrumented vehicle used in the testing campaigns.

This section aims at introducing the experimental setup used for the validation of both the Kalman filter and the Traffic Light Advisor system. The localization algorithm relies on a GPS receiver, responsible for locating the vehicle via latitude and longitude measurements, the integrated speed value available from the Electronic Control Unit (ECU) of the trolley-bus, and an Inertial Measurement Unit (IMU) that returns the values of acceleration along its axes. The main navigation system utilized is the GPS which is installed on the front part of the vehicle and provides spatial coordinates in a fixed reference frame. As mentioned, it can be missing for significant portions of the path when the number of satellites is not sufficient or the signal is not reliable, thus not allowing the algorithm to know the measurement of the vehicle’s position. The information coming from the GPS receiver must be then fused with other measurements coming from the ECU, providing the longitudinal velocity of the vehicle, and the 5 DoF IMUs measuring the acceleration of a body along the three main axes (*x*, *y*, *z*), as well as the rotational speed around the *x* and *y* axes as shown in Figure 1.

**Figure 2:**
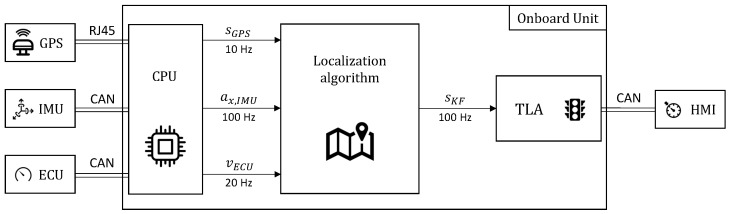
Architectural diagram of the vehicle’s sensor acquisition and processing.

It is worth mentioning that, as depicted in the architectural diagram in Figure 2, the available sensors have different sampling frequencies. In fact, the GPS rate is 10 Hz, while the ECU returns the speed value at 20 Hz and the acceleration from the IMU comes at 100 Hz. As a consequence, the localization algorithm running at 100 Hz has to deal with these different frequencies.

**Figure 3:**
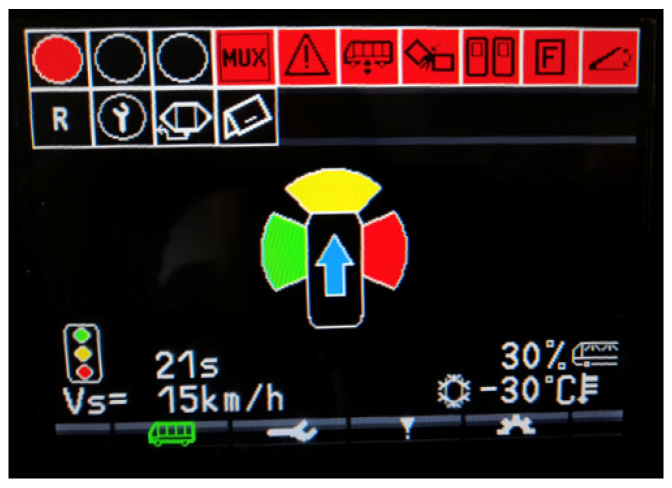
Example view of the Human–Machine Interface used for TLA experimental tests (snapshot with test values for all possible outputs).

On the onboard computational unit, the sensor acquisition, the localization algorithm, as well as the TLA system run on a soft real-time-based Robotic Operating System (ROS) architecture [32], allowing to have a simple framework for managing information coming from different sources using a publisher–subscriber logic. Within this framework, there are different nodes for publishing both the raw sensor data on the vehicle network and the vehicle data that can be read from its Controller Area Network (CAN), such as vehicle speed. This connection to the vehicle CAN-bus is used also to publish the outputs of the Traffic Light Advisor system so that the information can be shown to the driver on the integrated dashboard of the vehicle. Figure 3 reports a sample snapshot of the dedicated Human–Machine Interface (HMI). In particular, in the middle of the dashboard the driver receives a synthetic visual indication (i.e., an arrow indicator) to understand whether to accelerate or decelerate with respect to the current vehicle speed in order to reach the traffic light without stopping. This is done in order to minimize as much as possible the possible distraction source for the driver. However, additional information, such as the current traffic light status (i.e., top left corner), the time-to-change of the upcoming traffic light, and the value of the speed proposed by the TLA algorithm (i.e., bottom left corner), is provided in the periphery of the HMI.

**Figure 4:**
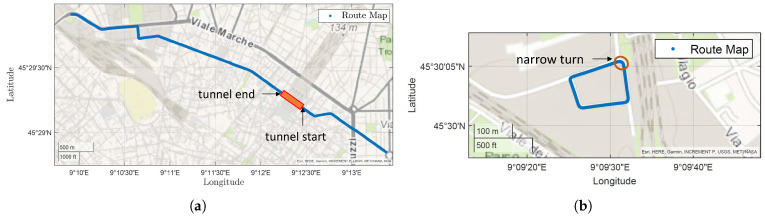
Testing areas in the city of Milan. (**a**) Testing Area 1; (**b**) Testing Area 2.

The testing area is available on a 4 km long portion of the regular service trolley-bus path in the city of Milan, being mainly covered in a preferential lane for public transportation. The route map (see Figure 4a) includes different scenarios, such as avenues with trees, urban canyoning, mid-narrow turns, and a tunnel 200 m long where the GPS is missing for a relatively long time. Furthermore, in order to have a better assessment of the localization algorithm, additional tests have been performed where a more favorable RTK correction for GPS is available, thus having a ground truth reference to evaluate the algorithm’s performances. Moreover, the second testing scenario (see Figure 4b) considered presents more severe testing conditions in terms of curve severity.

## 4. Localization Algorithm

**Figure 5:**
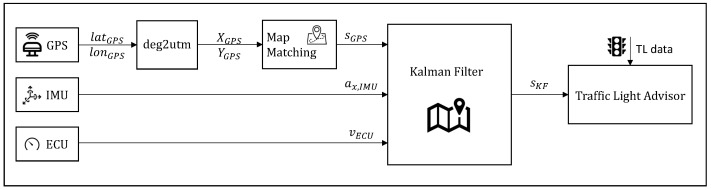
Scheme of the localization algorithm.

In order to minimize the error when estimating the position, a widely spread choice while dealing with linear systems is the use of the Kalman filter, also known as the Linear Quadratic Estimator (LQE). The principle of the Kalman filter (scheme in Figure 5) is to use a dynamic model of a system, with a number of variables constituting the state vector x and describing the system itself and its evolution over time. The system prediction step is taken thanks to known input variables, i.e., control inputs u, while the available measurements z are used to properly update the propagated variables in order to minimize the difference between the predicted states and the observed quantities. For the present application, the governing equations are those related to a simple uniformly accelerated 1D model:

(1) $$\left\{\begin{array}{c} s_{t+1}=s_{t}+v_{t}\;\cdot \;t+\frac{1}{2}a_{t}\;\cdot \;t^{2}\\ v_{t+1}=v_{t}+a_{t}\;\cdot \;t \end{array}\right.$$

where *s*, *v*, and *a* represent the vehicle’s position along the curvilinear coordinate on the path, speed, and longitudinal acceleration, respectively. The choice of such a simple model is justified by the fact that, on the one hand, the speed, and thus the accelerations, are limited. On the other hand, the TLA application needs just longitudinal accuracy along a predefined map of the path which has been obtained with the same algorithm presented in [33] based on the Cubic Hermite Spline (CHS).

Figure 5 summarizes the scheme of the implemented Kalman filter for localizing the trolley-bus along a known map of the route followed by the vehicle. In the following, the general mathematical description of the state estimator is reported, dividing the algorithm into four stages for the sake of clarity.

### 4.1. Process Equation

The first step reports the process equation, defining a discrete-time linear time-varying system as

(2) $$x_{k}=F_{k-1}x_{k-1}+G_{k-1}u_{k-1}+w_{k-1}$$

with it being possible to assume that the system matrices $F_{k-1}$ (i.e., state transition matrix), $G_{k-1}$ (i.e., control-input matrix), and control action $u_{k-1}$ are known without errors. In fact, according to Equation (1), the state transition and control-input matrices can be defined as

(3) $$F_{k-1}=\left[\begin{array}{cc} 1 & dt\\ 0 & 1 \end{array}\right]\;\;G_{k-1}=\left[\begin{array}{c} \frac{1}{2}dt^{2}\\ dt \end{array}\right]$$

where dt stands for the integration time step equal to 0.01 s.

The process noise $w_{k-1}∼\left(0,Q_{k-1}\right)$ assumes a random Gaussian zero-mean covariance $Q_{k-1}$, and it accounts for the noise related both to the modeling and to the input variables. The covariance $Q_{k-1}$ indicates how much the system model can be trusted for the prediction of the estimate. In fact, higher values of $Q_{k-1}$ indicate lower accuracy of the model, so less weight on the estimate.

In this work, the noise due to the model is assumed to be negligible and, as an additional assumption, the covariance matrix $Q_{k-1}$ is considered constant and diagonal:

(4) $$\begin{array}{cc} Q_{k-1} & =\left[\begin{array}{cc} Q_{pos} & 0\\ 0 & Q_{vel} \end{array}\right] \end{array}$$

in which the diagonal elements of the matrix are calculated as

(5) $$\begin{array}{c} Q_{pos}=dt^{2}\;\cdot \;\sigma_{a_{x}}^{2}\;\cdot \;dt^{2}=10^{-10} \end{array}$$

(6) $$\begin{array}{c} Q_{vel}=dt\;\cdot \;\sigma_{a_{x}}^{2}\;\cdot \;dt=10^{-6} \end{array}$$

where $\sigma_{a_{x}}^{2}$ represents the longitudinal acceleration variance, obtained from measurements.

### 4.2. Measurement Equation

**Figure 6:**
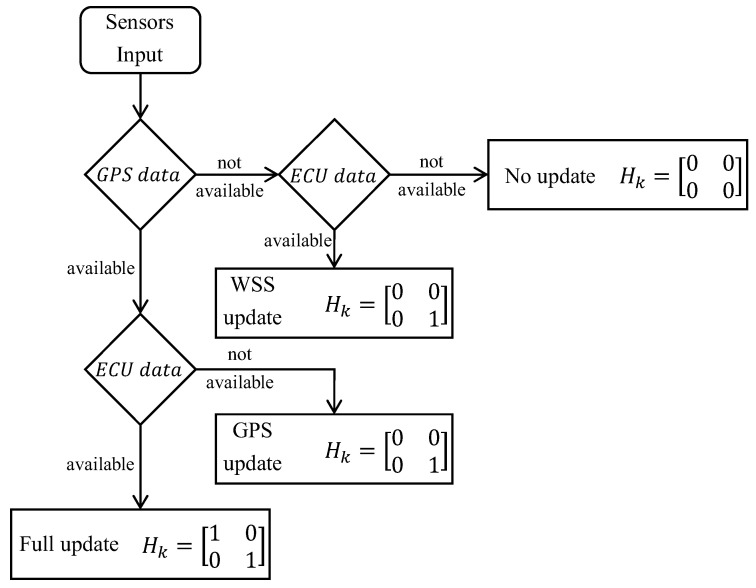
Measurement matrix $H_{k}$ workflow definition.

The measurement equation of the system is written as

(7) $$z_{k}=H_{k}x_{k}+v_{k}$$

where $H_{k}$ represents the measurement matrix structured as

(8) $$H_{k}=\left[\begin{array}{cc} H_{gps} & 0\\ 0 & H_{ecu} \end{array}\right]$$

with $H_{gps}$ and $H_{ecu}$ being Boolean values depending on GPS and ECU data availability, as depicted in Figure 6.

As far as the $H_{k}$ definition is concerned, in order to deal with the GPS outages and the multi-rate multi-sensor setup illustrated above, $H_{gps}$ and $H_{ecu}$ are Boolean variables defined depending on each sensor’s availability at time *k*. Both $H_{gps}$ and $H_{ecu}$ are initially set to zero; if a GPS measure is available and it is considered reliable as the number of satellites received is greater than 7, then $H_{gps}$ is set to 1. As for the speed, if a new speed measurement arrives, $H_{ecu}$ is set to 1. It is worth noting that the speed value read from the ECU is considered always reliable, as the vehicle is thought to run in a standard adherence condition with a limited average speed. In case no measurement is available for the update, $H_{k}$ remains null; thus, the state keeps on just being predicted. Performing this check every 0.01 s, the consequence, in the best-case scenario, is that the GPS update occurs just once every 10 time steps, while the ECU update happens once in 5 time steps.

Gaussian measurement noise $n_{k}∼\left(0,R_{k}\right)$ is associated with the sensors used for the measurement update. It is characterized by zero mean and covariance $R_{k}$ defined as

(9) $$R_{k}=\left[\begin{array}{cc} R_{gps} & 0\\ 0 & R_{ecu} \end{array}\right]$$

where $R_{gps}=10^{-1}$ and $R_{ecu}=10^{-5}$ are tuning parameters for the filter related to the reliability of the sensors, as they are obtained by computing the variance of the signals of the two sensors. In fact, more reliable sensors lead to lower $R_{k}$ values, while sensors introducing more noise will be responsible for higher $R_{k}$ values, thus leading to a lower impact of the measurement update on the state estimation. These values are then adjusted to obtain additional stability for the estimate, especially when the vehicle stands still. The idea behind the tuning is based on the following principles:

- $R_{gps}>R_{ecu}$ in the driving condition: the availability and the accuracy of the GPS depend on many different factors, while the Wheel Speed Sensor is much more reliable and accurate, as the values of the variance confirm;
- $R_{gps}>>R_{ecu}$ in the standing-still condition: the value of $R_{gps}$ has been increased to 1 when the vehicle speed is lower than 1 km/h; otherwise, the covariance values remain the default ones. In this way, when the vehicle stands still, the GPS data are less considered as they are less reliable and accurate for the update, while the ECU speed value becomes much more important.

### 4.3. Time Update Equations

In the prediction step defined in (2) at time *k*, the predicted state ${\hat{x}}_{k}^{-}$ and corresponding covariance $P_{k}^{-}$ are calculated according to the model:

(10) $$\left\{\begin{array}{c} {\hat{x}}_{k}^{-}=F_{k-1}{\hat{x}}_{k-1}^{+}+G_{k-1}u_{i-1}\\ P_{k}^{-}=F_{k-1}P_{k-1}^{+}F_{k-1}^{T}+Q_{k-1} \end{array}\right.$$

### 4.4. Measurement Update Equations

When the measurements are available at instant k, then the measurement step consists of the following equations:

(11) $$\left\{\begin{array}{c} K_{k}=P_{k}^{-}H_{k}^{T}\;{\left[H_{k}P_{k}^{-}H_{k}^{T}+R_{k}\right]}^{-1}\\ {\hat{x}}_{k}^{+}={\hat{x}}_{k}^{-}+K_{k}\;\left[z_{k}-H_{k}{\hat{x}}_{k}^{-}\right]\\ P_{k}^{+}=\left[I-K_{k}H_{k}\right]\;P_{k}^{-} \end{array}\right.$$

These equations represent the updated estimations for the state ${\hat{x}}_{k}^{+}$ and the covariance $P_{k}^{+}$, respectively, and these two quantities will be required for the following step k+1 in the prediction step equations shown above.

## 5. Traffic Light Advisor Experimental Validation

The Traffic Lights Advisor (TLA) system is an auxiliary tool for the driver, which is able to provide real-time information about the following traffic light’s phase while suggesting the speed to cruise through the intersections on the path during the green light phase. Entering into the details of the TLA as an ADAS, it is expected to deal with the typical situations faced approaching a traffic light:

- Stop&Go: the algorithm is intended to properly modulate the vehicle speed in order to avoid a complete stop (when possible) in front of the traffic light.
- Last-second braking: the algorithm should inform the driver about the need to slow down, as an acceleration maneuver is not feasible.
- Unnecessary stop: the algorithm aims at suggesting to the driver the recommended speed (compliant with road limits and vehicle safety) in order to pass the upcoming intersections during the green phase of the traffic light.

As a result, the main goal of this algorithm is to save both travel time and the energy used by the vehicle. This is done by considering the 4 traffic lights ahead on the path closest to the vehicle. In particular, the algorithm is thought to consider a uniformly accelerated motion model for the vehicle to reach the suggested speed within the first traffic light of the series and then keep that velocity to safely pass the upcoming intersections without stopping. In the following, the most relevant features of the functioning of the TLA system are reported; for further details, the reader can refer to the work in [6] where the full design of the algorithm is presented.

The TLA system is based on an iterative algorithm for selecting the recommended velocity which has to fulfill the following constraints:

- The velocity has to be below the maximum allowed speed limit for the road.
- The vehicle should be able to reach the target velocity following a uniformly accelerated motion model within the end of the first available green phase of the first traffic light ahead, with the the maximum vehicle acceleration limited to 1 $m/s^{2}$ for both safety and comfort concerns.
- Considering the generic *i*th traffic light, the vehicle’s admissible speed range is obtained from the intersection between the required velocity to reach the upcoming intersection and the admissible speed range computed for the (i−1)th traffic light.

**Figure 7:**
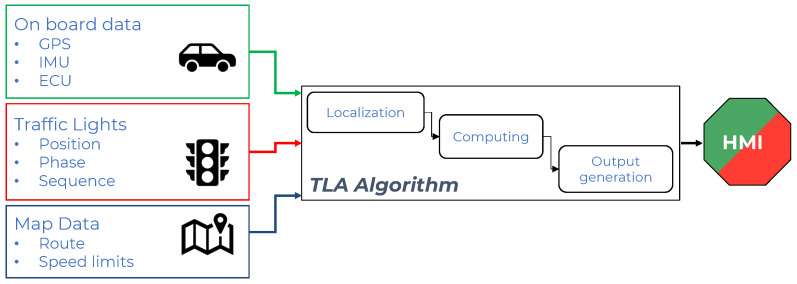
Schematics of the Traffic Light Advisor algorithm.

Figure 7 represents the scheme of the Traffic Light Advisor system: the inputs, coming from onboard sensors for the localization algorithm presented in Section 4; the traffic lights; and the map enter the algorithm that produces an output that is shown in the HMI.

As far as the traffic light data are concerned, through the municipality of Milan there has been the possibility to know the traffic light plans of the intersections along the route. These data are fed to a “Traffic Light Generator” which is in charge of emulating the real traffic lights on the road. This emulator, implemented in the Matlab-Simulink environment, communicates the traffic lights’ phases, sequences, and positions to the TLA algorithm.

In this work, the aim is to add an experimental validation of the non-optimal Traffic Light Advisor algorithm presented in [6]. To validate the functioning of the algorithm, similarly to what is performed in [34], three different cases are considered:

- **Base Case**: test run without the TLA used to establish a benchmark. This is obtained by letting the driver behave as usual, with no additional information available to the driver with respect to the typical driving case.
- **TLA Case**: test run with the TLA running and showing information to the driver who tries to follow the instructions. The algorithm, running in real time, conveys the information to the driver through a specifically designed HMI (see Figure 3).
- **Ideal Case**: simulation run in post-process on the basis of the TLA Case data experimentally acquired, aiming to assess the behavior of the algorithm, assuming an ideal driver able to perfectly follow the algorithm’s instructions. This is proposed to check whether the algorithm is working correctly, observing how it would operate if no constraints set by external factors such as traffic, the driver’s reflexes, and other interference were to impact its ideal behavior.

In Section 6, the results of the experimental campaign are reported, proposing a comparison between the Base Case and TLA Case. Subsequently, the real test performed on the road is compared with the aforementioned Ideal Case. The comparison is carried out not only by looking at the kinematic performances in terms of the covered distance, average speed, and acceleration but also from an energetic point of view. In fact, knowing the vehicle mass (i.e., m= 19,800 kg) and the vehicle acceleration *a* and velocity *v* from the onboard sensors, it is possible to obtain a rough evaluation of the power consumption expressed in (kW) starting from the inertial force as

(12) $$P=\frac{m\;\cdot \;a\;\cdot \;v}{1000}$$

From the power consumption, it is possible to derive then the following index, i.e., Instantaneous Energy Consumption (IEC) expressed in (kWh/100 km), which allows quantifying the energy being drained by the vehicle:

(13) $$IEC=P\;\frac{\Delta t}{\Delta s}$$

where Δt is the time step of the algorithm and Δs is the distance covered by the vehicle in the time span.

## 6. Results

This section is devoted to the presentation of the validation results of both the localization state estimator and the Traffic Light Advisor algorithm. The tests have been performed on two different paths, as shown in Figure 4, because of the availability of the traffic light information during the test runs.

### 6.1. Localization

The state estimator presented in Section 4 runs at 100 Hz as a standalone C++ ROS node generated in the Matlab-Simulink environment [35], subscribing to the sensors’ topics present in the ROS network of the vehicle. In the following, the results of the state estimator experimental data on the two Testing Areas are reported. It is worth noticing that the measurement used by the Kalman filter is the raw data of the GPS device, as it represents a more general and applicable condition in urban scenarios. As far as the localization accuracy is concerned, the estimation error is evaluated using as ground truth the data coming from the real-time kinematic (RTK)-corrected GPS data which reaches cm level precision.

The analysis is focused on three main conditions:

- Vehicle standing still: this condition is quite frequent in urban scenarios because of the high number of intersections and the stops along the path of a local public transportation vehicle. The typical GPS behavior in this situation is to fluctuate around the actual position, causing the vehicle localization to change, both forward and backward.
- Prolonged GPS outage: this condition is usually faced because of urban canyoning, high trees, or tunnels.
- Regular driving with curved path: this is the general scenario to be considered for the localization accuracy assessment.

**Figure 8:**
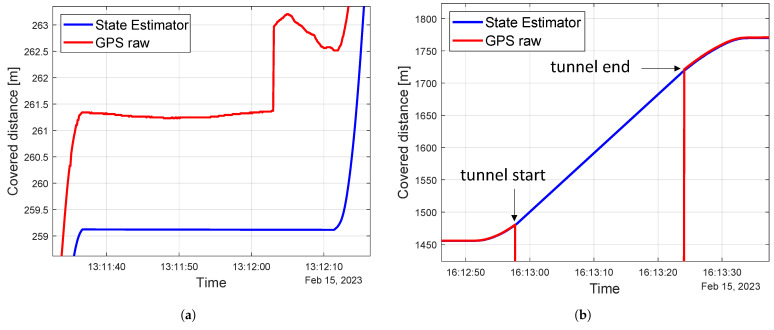
Testing Area 1: state estimator results analysis. (**a**) Vehicle standing still; (**b**) long GPS outage.

The first two items are analyzed in Testing Area 1 (i.e., Figure 4a) which covers the regular service path of the vehicle. Figure 8a depicts the trend of the covered distance (i.e., the curvilinear coordinate along the path) in correspondence to a stop. As can be seen, on the one hand, the GPS trend is floating; on the other hand, the state estimator is able to provide a constant *s* value thanks to the increase in the $R_{gps}$. This allows having a constant value for the curvilinear coordinate when the vehicle is stopped, which is fundamental for the TLA application as the change in the distance of the traffic light ahead affects the TLA speed calculation. The plot reported in Figure 8b shows the state estimator trend during a prolonged GPS outage because of a 200 m long tunnel. The distance between the state estimator value $s_{KF}$ and the GPS coordinate $s_{GPS}$ as soon as it becomes available is equal to 1.86 m. Although this value is relatively high, the GPS benchmark after a long outage has to be considered as not reliable, as it requires some time (i.e., 20–30 m) to obtain acceptable GPS accuracy.

The testing campaign on the second Testing Area (i.e., Figure 4b) aims to evaluate the actual localization accuracy that can be obtained with the proposed localization algorithm. In fact, this area is characterized by better GPS coverage as there are no urban canyons that typically affect the GPS signal. Furthermore, the route path has four narrow turns, allowing to assess the behavior of the state estimator in the turn condition as well. In this case study, the raw data of the GPS, i.e., before the RTK correction, are fed to the localization algorithm. The state estimator output is then compared with the RTK correction GPS data which provides the ground truth value for the accuracy assessment.

**Figure 9:**
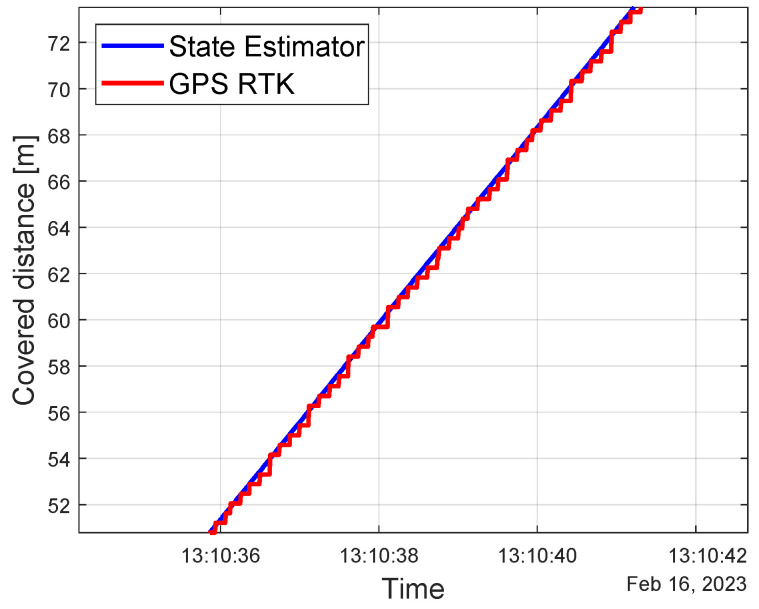
Testing Area 2: state estimator result analysis.

**Figure 10:**
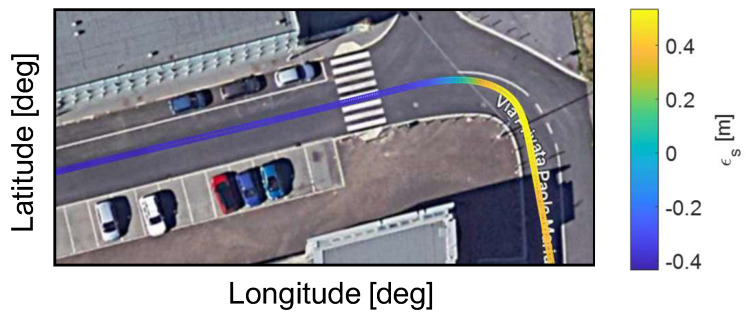
Testing Area 2: state estimator accuracy in curve condition.

In Figure 9, a portion of the trend of the covered distance on the closed path of the Testing Area 2 is shown. In this scenario, it is possible to appreciate a much smoother trend of the state estimator with respect to the step-wise GPS signal because of the higher frequency of the localization algorithm, i.e., 100 Hz. When considering the entire path, the estimation error can be calculated as

(14) $$\epsilon_{s}=s_{RTK}-s_{KF}$$

where $s_{RTK}$ and $s_{KF}$ are the curvilinear coordinate along the map of the GPS RTK corrected and the proposed Kalman filter, respectively. Taking the Root Mean Square value of the error trend over the whole path, it turns out to be equal to 0.28 m. Figure 10 reports the trajectory of the vehicle estimated by the proposed Kalman filter in the narrowest turn on the path. The color indicates the estimation error $\epsilon_{s}$, with the estimation error limited to 0.4 m also in the turn condition.

The obtained experimental results show good accuracy for the TLA application, which just needs the vehicle location along the path to compute the distance from the upcoming traffic lights. Furthermore, as the vehicle considered is a large local public transportation vehicle, changes in speed and direction are typically not so harsh, so the choice of a one-dimensional model, on the one hand, allows to minimize the implementation effort. As far as the behavior in the curve condition is concerned, the results are promising and allow running the TLA algorithm in a general urban scenario, although the model appears to not be accurate enough for control logic dealing with vehicle lateral dynamics.

### 6.2. Traffic Light Advisor

**Figure 11:**
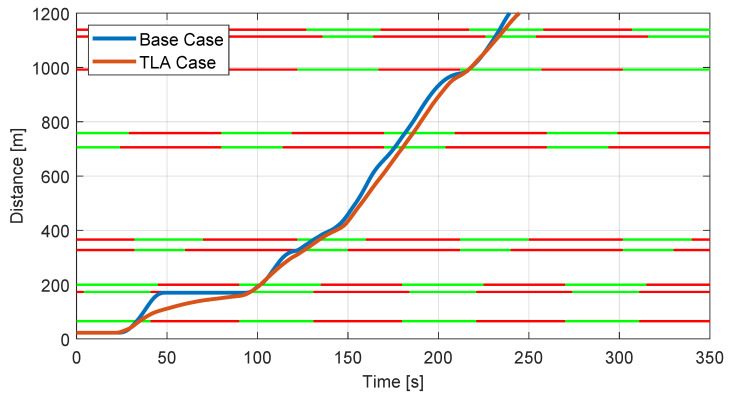
TLA Case comparison with respect to Base Case.

As mentioned in Section 5, the assessment of the TLA performances is performed both from a kinematic and an energetic point of view. In particular, in Figure 11, the plot of the covered distance of one test run, comparing the Base Case of the driver with the TLA Case, is shown. The horizontal lines represent the traffic lights status on the path, with the sequence between the red and green phases. It is worth mentioning that the yellow phase has been included in the red one for adding an extra safety margin to the algorithm.

From the graph, it is possible to observe how the TLA system, besides avoiding the stop at the second traffic light encountered by the vehicle, allows having a smoother trend for the entire path, meaning that the velocity has a lower fluctuation, and thus lower acceleration.

Table 1 proposes a similar analysis from a quantitative point of view, showing the Root Mean Square values of velocity, acceleration, and IEC, previously defined. The results indicate that, when the TLA is active, the vehicle proceeds with a lower average speed with respect to the Base Case. This can sound unexpected, but it is consistent with the lower acceleration value, as the driver tends to accelerate more than required to cruise through all the intersections without stops. Besides more comfort for passengers, having lower accelerations guarantees lower energy consumption, with a 40% reduction in the energetic indicator IEC when the TLA is active. Furthermore, these values confirm the results obtained from the simulations conducted in [6].

**Figure 12:**
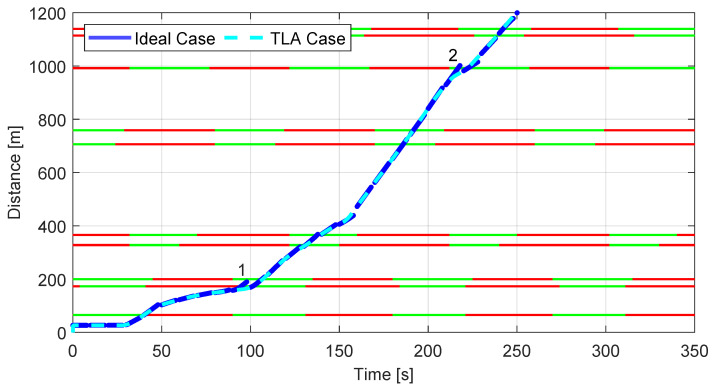
TLA Case comparison with respect to Ideal Case.

The plot in Figure 12 is intended to investigate in which situation the algorithm has an improvement margin and how it could be adjusted to further enhance its impact.

In fact, the spotlight is on the algorithm’s reaction to the inability of the driver to perfectly follow the speed reference. The plot reports the previously shown trend of the TLA Case and it is compared with a set of ideal behaviors of the vehicle obtained by running different simulations 8 s long, each one having the vehicle position and velocity in correspondence to the start of the simulation (i.e., current actual vehicle position and velocity) as the initial conditions. This allows to see how the vehicle would have proceeded in an ideal case, thus highlighting, on the one hand, the effect of external factors as well as driver behaviors, and, on the other hand, the capability of the TLA system to adapt to the conditions the vehicle is facing. Two main points of interest are indicated in the plot, those in which the ideal response to given real conditions (i.e., blue solid line) is suggesting a different behavior. In both cases, the vehicle should accelerate right before crossing the facing traffic light, but the driver is more confident in waiting for some seconds because the traffic in front of the vehicle is still showing a red light. This is a natural tendency of human drivers to not fully trust an indication of the HMI. In fact, although based on actual real-time data about the traffic light time-to-change, in some cases the TLA is perceived unsafe as it may also suggest accelerating when a common driver, without knowing when the light is going to change, would not.

## 7. Conclusions

In this work, a localization algorithm based on Kalman filtering for a Traffic Light Advisor application and the TLA experimental validation are proposed. The implemented Kalman filter is designed to run at 100 Hz with a simple 1D kinematic model and measurements from sensors having different sampling frequencies. Real-world tests provided results accurate enough to be integrated into the TLA algorithm with an average error lower than 0.5 m, having robust behavior both in long GPS outage situations and standing-still and curve conditions thanks to the filter weight tuning and map matching. Regarding the TLA validation, the experimental campaign confirmed the positive impact on both comfort, service regularity, and energy consumption with respect to unassisted driving. The comparison with a simulated ideal case highlighted the areas of improvement for the actual implementation of the system, such as the presence of traffic in front of the vehicle, and external factors, like road unevenness, that make the driver slow down, as well as driver difficulties in following and trusting the HMI indications.

As future developments, on the one hand, it would be interesting to extend the state estimator to a 2D vehicle model, aiming at using the implemented localization algorithm for other ADAS applications also considering later dynamics of the vehicle. On the other hand, the TLA experimental validation would need additional testing campaigns to perform a larger statistical assessment of the algorithm. Furthermore, it would be valuable to introduce information about the traffic ahead in the TLA algorithm to cope with the challenges that emerged from the test.

## Figures and Tables

**Table 1:** RMS values for speed, acceleration, and IEC: comparison between Base Case and TLA Case.

	Base Case	TLA Case	Reduction
$v_{RMS}$ [m/s]	6.50	4.95	−23.7%
$a_{RMS}$ [m/s${}^{2}$]	0.53	0.31	−44.3%
$IEC_{RMS}\;\left[\frac{\mathrm{kWh}}{100\;\mathrm{km}}\right]$	291.39	175.92	−39.6%

## Data Availability

Not applicable.
